# Key Features in the Design and Function of Nanocarriers for Intranasal Administration of Gene Therapy in Huntington Disease

**DOI:** 10.33696/nanotechnol.4.043

**Published:** 2023

**Authors:** Oksana Fihurka, Stephen Aradi, Vasyl Sava, Juan Sanchez-Ramos

**Affiliations:** 1 Department of Neurology, University of South Florida, USA

**Keywords:** Gene therapy, Huntington’s Disease, Intranasal administration, Nanocarriers

## Abstract

A major obstacle to fulfilling the therapeutic promise of gene therapies for hereditary brain diseases, such as Huntington’ Disease (HD), is the requirement for viral vectors and/or an invasive delivery system (stereotaxic injection into brain or infusion into the intrathecal space). HD is an autosomal dominant neurodegenerative disease for which several clinical trials have demonstrated gene-lowering effects following intrathecal administration. These technical limitations have given impetus to the development of alternative non-invasive delivery systems for gene therapy of brain diseases. The overall objective of this review is to discuss the key features in the design of nanocarriers for intranasal administration of gene-therapy for HD, focusing primarily on our series of published work on the use of nanocarriers for gene therapy. Design and development of nanocarriers packaged with gene-lowering agents represents a significant advance towards non-invasive nose-to-brain delivery of gene therapy for HD and other hereditary brain disorders.

## Graphical Abstract

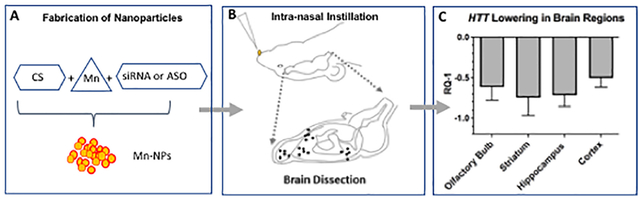

## Introduction

The control of gene expression by therapeutic agents has great potential for the field of neurological diseases. Such therapeutic approaches include the suppression of expression of toxic gain-of-function mutations, or the introduction of genes expressing constituents that are beneficial to a particular condition. Gene expression can be attenuated by several approaches, including nucleic acid-based therapies with antisense oligonucleotides (ASOs) or RNA interference (*RNAi*), via small interfering RNA (*siRNA*) or micro-RNA (*miRNA*). Currently, nucleic acid therapies may be delivered via intermittent administration intrathecally or via intracerebroventricular injection or may be introduced by intraparenchymal injection of viral vectors encoding these therapies. *In vivo* delivery of ASO or RNAi has been shown to be effective in animal models of Alzheimer’s disease (AD), amyotrophic lateral sclerosis (ALS), spinocerebellar ataxia (SCA), and HD [1–2].

HD is an autosomal dominant hereditary disease caused by an expanded trinucleotide (CAG) tract in exon 1 of the huntingtin gene (*HTT*) [3]. The mutated *HTT* gene encodes a protein, mutant huntingtin (mHtt), characterized by a long polyglutamine tract. The mHtt protein misfolds, accumulates in neural tissues and causes neuronal dysfunction and neurodegeneration at all levels of the central nervous system (CNS) [4]. Clinically, patients experience a gradually worsening set of motor, cognitive and psychiatric symptoms that inexorably results in death. Management at present is solely symptomatic.

Since the discovery of the pathologic *HTT* mutation in 1993 and the creation of animal models of the disease, novel therapies have been developed to reduce expression of the *HTT* gene – so-called huntingtin-lowering therapies [3,5–8]. Repeated intrathecal injections of an anti-*HTT* ASO (*Tominersen*), was reported to significantly reduce concentrations of htt protein (both normal and mutant htt) in the CSF of HD subjects [9]. While this was recapitulated in a subsequent global Phase III randomized, placebo-controlled trial, dosing was stopped early, on the recommendation of the Independent Data Monitoring Committee (IDMC) [10]. The IDMC reported lack of benefit and poorer clinical outcomes compared to placebo treatment. They also found increased adverse events in the highest treatment dose arm [11,12]. Several explanations for these findings have been proposed including a) the possibility that the ASO infused into the cerebro-spinal fluid (CSF) by intrathecal injections did not attain adequate levels in critical brain regions such as corpus striatum, b) treatment-related central nervous system (*CNS*) inflammation as evidenced by elevated CSF white blood cell counts and protein concentrations, and c) inflammatory or otherwise off-target neural injury as indicated by transient increases in neurofilament light protein in treated subjects. In addition, the neuro-inflammatory changes triggered by the mutant protein itself [13] continue unabated resulting in disease progression despite robust huntingtin lowering. Going forward, non-invasive gene therapies will need to be developed that both lower m*HTT* expression and suppress neuro- inflammatory processes.

Polynucleotides (*ASO, siRNA*) and proteins have a significant limitation as neurotherapeutic agents for brain disease because they do not readily cross the blood-brain barrier (*BBB*) and cannot survive intact in the gut or blood. As a result, gene therapy for brain disorders has required direct neurosurgical microinjection or infusion into brain or cerebrospinal fluid. Recent research suggests intranasal administration is a viable route of administration for brain disorders. Researchers have reported direct nose-to-brain delivery of relatively large molecules, including neurotrophins (*NGF and insulin-like growth factor [IGF]-1*), neuropeptides, cytokines (*interferon β-1b and erythropoietin*) as well as polynucleotides (*DNA plasmids and genes*) [14–20]. In a most recent example, compacted DNA nanocarriers (*encoding a reporter gene, eGFP*) were successfully delivered from nose to brain in rats without the need for viral vectors [15].

Nanocarriers have been designed in our laboratory and developed for nose-to-brain delivery of gene-silencing agents such as ASO or siRNA. These nanocarriers demonstrate the ability to protect the nucleic acid cargo from degradation, and to facilitate cellular entry [21]. The current gene therapies for brain disease are invasive, requiring intrathecal or direct intracerebral injection. The intranasal approach for drug delivery to brain is easy to administer, well-tolerated and permits safe, chronic intermittent administration of gene-silencing agents. Moreover, the nose-to-brain delivery system does not require viral vectors which can trigger encephalopathy in some patients. Another issue to be addressed relates to the chronic neurodegenerative process triggered by accumulation of mutant htt protein which continues to progress even when *HTT* expression is lowered. This problem was underscored by observations in the Tominersen study which revealed lowering *HTT* expression was insufficient for slowing clinical progression in older patients with a higher disease burden [10].

In this review, the key characteristics for the design and function of nanocarriers for intranasal administration of gene therapy of HD will be identified and discussed. The ultimate goal of this research will be to encourage other researchers to translate pre-clinical data on optimized nanocarriers to clinical trials in patients with HD.

## Size-controlled Enrichment of Nanocarriers

The size of nanocarriers is a critical variable that impacts circulation half-life, extravasation, and macrophage uptake [22]. The nanocarrier should not be too small, which would result in rapid clearance from the body. An excessively large size would increase capture of the nanocarriers by macrophages resulting in decreased availability for the target tissue. A nanocarrier below 10 nm in diameter is prone to clearance through renal excretion. The largest nanocarrier applicable for drug delivery must be capable of penetrating permeable vasculature for the successful delivery of drugs to the target tissue [22]. The gap junction for the endothelial cells in leaky vasculature ranges from 100–600 nm [23]. Therefore, an effective nanocarrier size is from 10 to 200 nm to ensure longer circulation time and increased accumulation in target tissue.

Nanocarrier size is also important for effective release of payload at the target tissue. Smaller nanocarriers have been shown to release their payload at a faster rate than larger nanocarriers. Larger nanocarriers, despite their slower release of payload, were capable of releasing larger numbers of packaged molecules [22].

**Figure 1.**
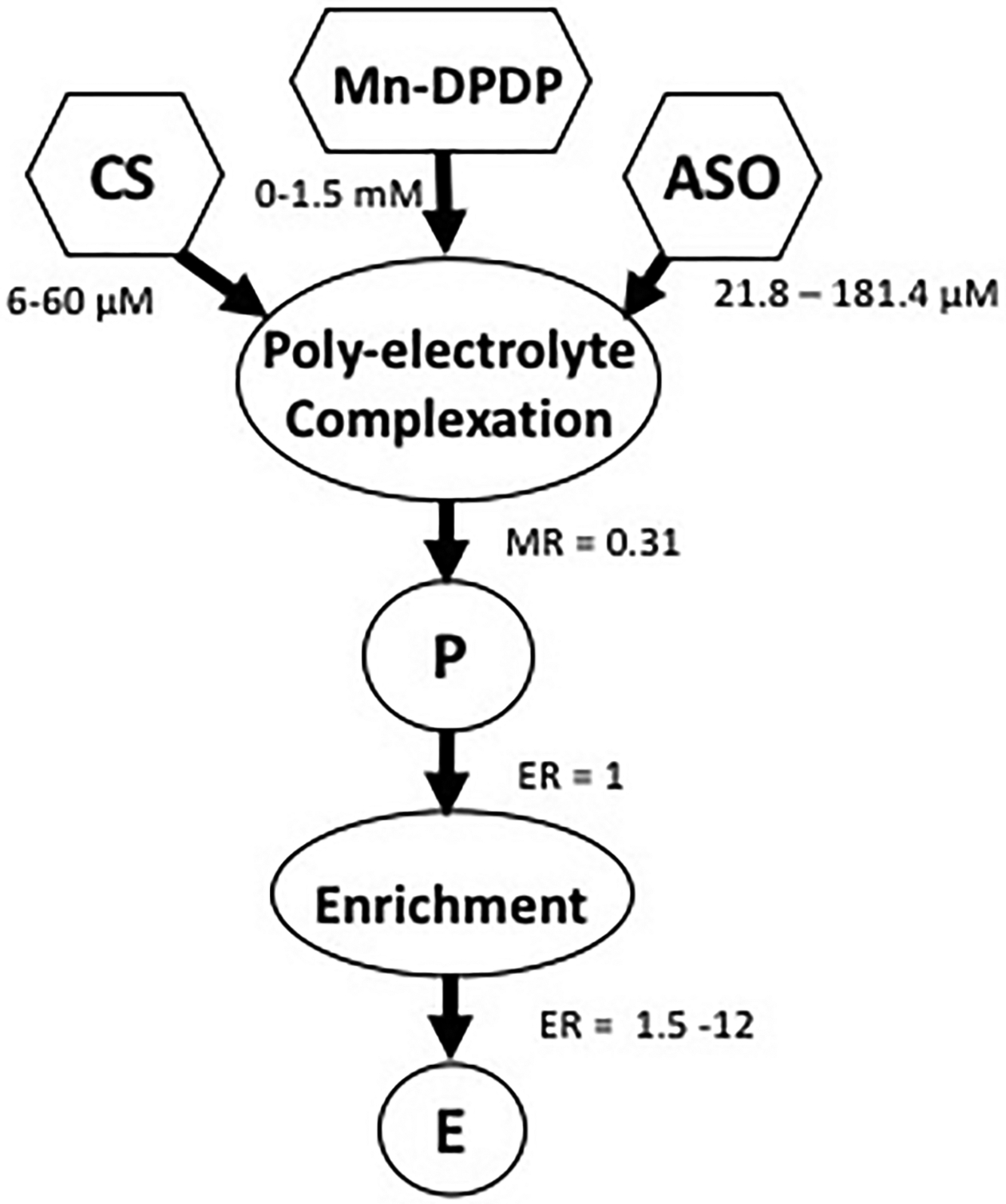
Schematic of nanocarrier fabrication. Previously published by Sava et al. [24] (Copyright Elsevier, 2020).

Figure 1 shows the flow chart of a two-step method for fabrication of nanocarriers by loading chitosan-based nanoparticles with siRNA. The nanocarriers were made by polyelectrolyte complexation of diluted ingredients which include small interfering RNA (*siRNA*), Mn-Dipyridoxal diphosphate (*Mn-DPDP*) as a crosslinker and chitosan (*CS*). The provisional nanocarriers (P) were then concentrated to produce an enriched preparation (*E*). The polyelectrolyte complexation reaction between chitosan and siRNA was performed at concentrations that yield a particle size of around 100 nm. The enriched preparation (*E*) was prepared to obtain increased concentration of nanocarriers to deliver the required dose via intranasal administration.

**Figure 2.**
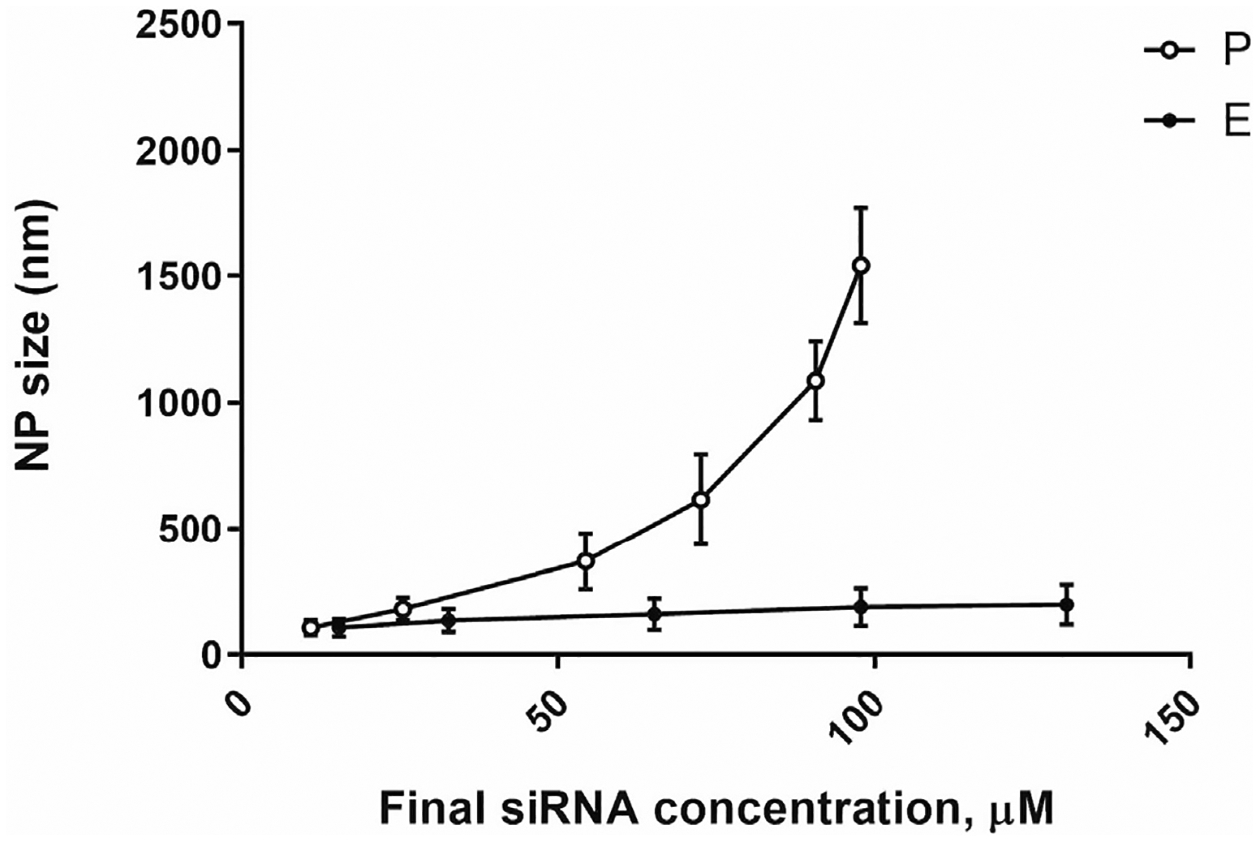
Changes in NP (nanocarrier) size for P (circles) and E (squares) preparations as a function of siRNA concentrations. Previously published by Sava et al. [24] (Copyright Elsevier, 2020).

This two-step approach resolves the limitation of the polyelectrolyte complexation procedure. Experimental results indicate that increasing concentrations of siRNA in the polyelectrolyte complexation reaction result in exponential growth of nanoparticle size (Figure 2). Thus, reliable fabrication of chitosan-based nanocarriers is possible only in diluted concentrations of all components. However, the resultant increased volume of preparation limits its utility for intranasal administration. To lower the volume of nanoparticle preparation for obtaining the necessary dose of siRNA for intranasal administration, the provisional preparation (*P*) was subjected to enrichment based on centrifugal evaporation of water. It was reported that the enrichment protocol can provide up to 12-fold increased concentrations of siRNA in the nanocarrier preparation [21].

**Figure 3.**
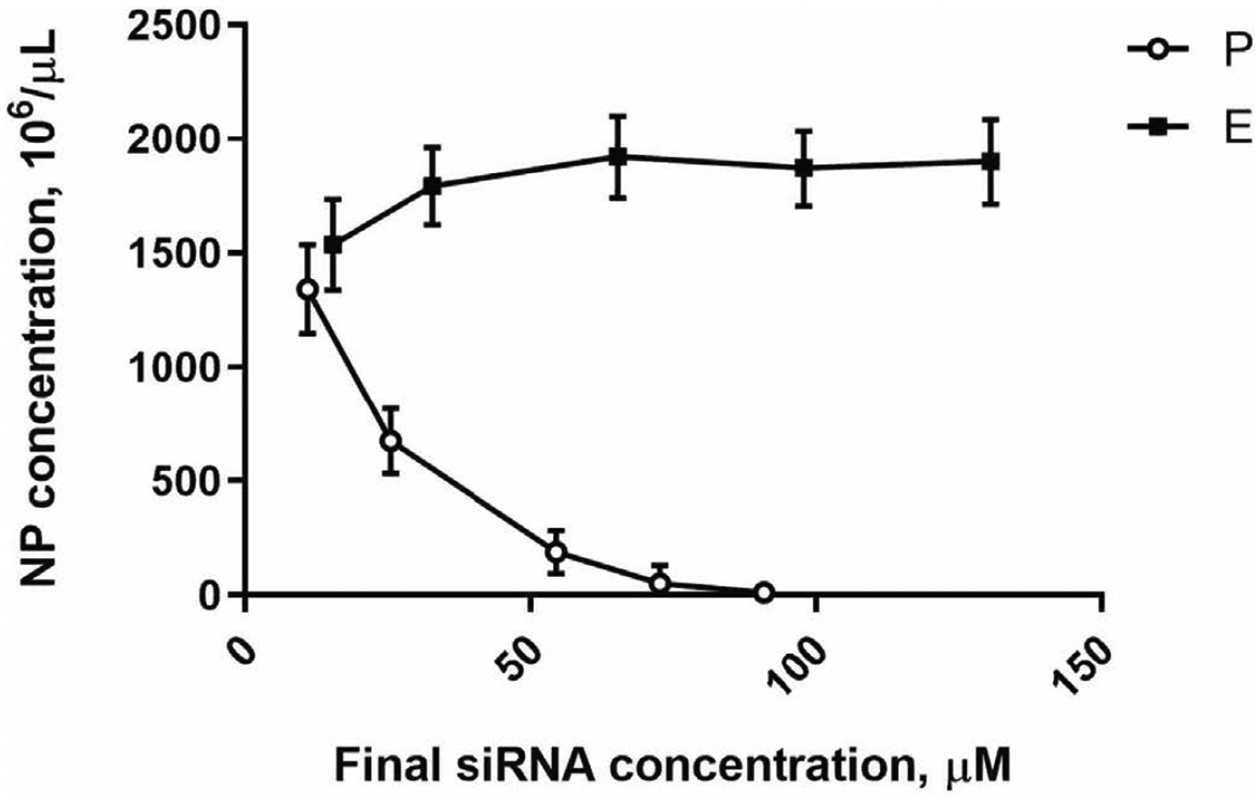
Nanoparticle (nanocarrier) concentrations in P (circles) and in E (squares) preparations as a function of siRNA concentration. Previously published by Sava et al. [24] (Copyright Elsevier, 2020).

Notably, the number of nanocarriers per volume (NP concentration) decreases as concentration of ingredients for the complexation reaction increases. Figure 3 shows changes in NP concentrations for both provisional and enriched preparations depending on siRNA content. Increasing the concentration of siRNA in the complexation reaction negatively affects NP concentration due to the exponential growth in size of NP (Figure 2). Enrichment allowed higher concentration of siRNA without substantial change in the NP size.

**Figure 4.**
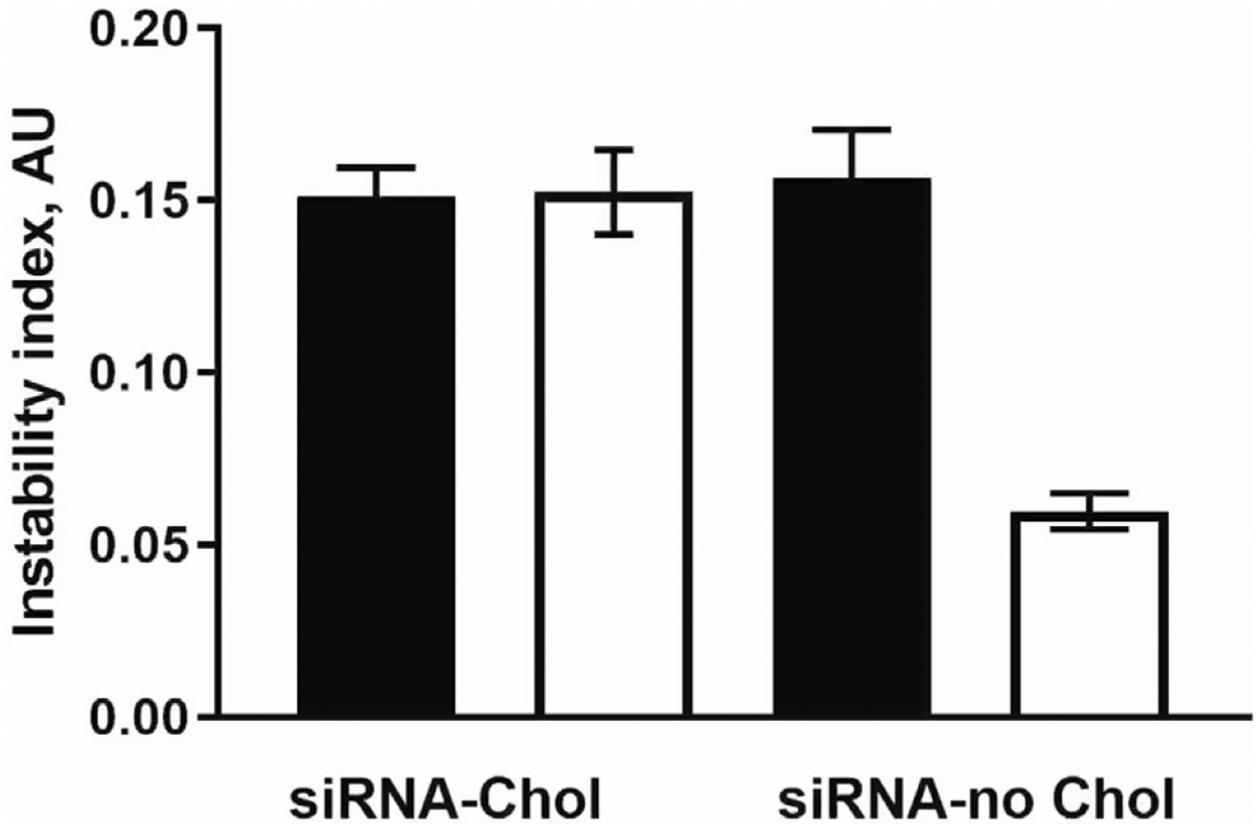
Comparison of the physical instability of chitosan nanoparticles containing cholesterol- siRNA (siRNA-Chol) to nanoparticles loaded with siRNA free of cholesterol (siRNA-no Chol). Closed and open bars represent P and E preparation, respectively. Previously published by Sava et al. [24] (Copyright Elsevier, 2020).

Even as enrichment increases siRNA dose delivered via intranasal administration of nanocarriers, the enrichment may introduce some instability in the preparation, depending on lipophilicity or hydrophilicity of the cargo molecule. The enriched nanocarriers became even more stable as compared to the provisional preparation when using a more hydrophilic siRNA (*with no conjugated cholesterol*). This is an important factor to consider when fabricating nanocarriers. The effect of enrichment on physical instability of NP in association with siRNA lipophilicity is shown in Figure 4. The instability index expressed in arbitrary units (*AU*) ranged from 0 (*most stable*) to 1 (*most unstable*).

Several double stranded RNA oligonucleotides were packaged into nanoparticles. These were synthesized at the University of Massachusetts (UMASS) RNA Institute [24,25]. There were two types of siRNA designated for lowering expression of the *HTT* gene. One of them (cy3 HTT 10150-P2VP- Chol) contained cholesterol conjugated at the 3’ end of antisense strain. Another one (*cy3 HTT 10150-P2VP*) has 3’-end free.

Chitosan has properties that make it useful for packaging nucleic acids like siRNA. Chitosan polymerizes to form compact nanocarriers due to electrostatic interactions between positive charged moieties of its amino groups and negative charged phosphate moieties of the siRNA structure. The Mangafodipir was used as a crosslinking agent to stabilize the globular structure of nanocarriers and protect siRNA from degradation. Another advantage conferred by using a chitosan matrix is that it does not bind the siRNA too tightly, thereby allowing release of siRNA to participate in gene silencing. In addition, Mn-containing NPs can be visualized in MRI T1-weighted imaging, allowing the researcher to track and quantify the transport and distribution of the NPs to brain [26].

Other matrices in addition to chitosan have been studied [21]. However, the application of chitosan has multiple advantages. Chitosan is readily biodegradable by lysozymal enzymes and has little or no toxicity. Chitosan/siRNA complexes form nanocarriers with dimensions appropriate for intranasal delivery. Another key feature is that structurally intact siRNA is released from the chitosan- based nanocarriers, an essential prerequisite for nanocarrier-mediated RNA gene silencing.

To summarize, fabrication of nanocarriers of relatively small size (*100–160 nm*) is an important factor for successful intranasal delivery of payloads to brain. The nature of interaction between siRNA and chitosan requires that fabrication of the nanocarriers occur in relatively diluted concentrations, which limits intranasal dosing due to the large volumes required to deliver an effective dose. Therefore, the ability to fabricate concentrated nanocarrier preparations without damaging siRNA content is a critical factor for successful intranasal delivery of gene silencing agents for neurodegenerative diseases such as HD.

## Kinetic Control of Repetitive Intranasal Dosing

Chitosan NPs can effectively deliver siRNA cargo into mouse brain via intranasal administration [21,25,26]. The kinetics of consequent lowering of target gene expression is important for determining dosing frequency and intervals that would be expected to achieve a therapeutic magnitude and duration of effect. Prior reports have described such kinetics, but none have examined an intranasal delivery route [27–30]. We sought to develop a mathematical model of the kinetics of our intranasally delivered nanocarrier and compare this to *in vivo* performance in a transgenic mouse model of HD (*YAC 128 mice bearing human mHTT)*. In light of the limited duration of gene silencing produced with a single dose, repetitive dosing is required to maintain sustained lowering of gene expression.

The effects of intranasal dosing intervals on the magnitude and duration of *HTT* gene lowering in various brain regions was recently examined in YAC 128 transgenic mice [30]. The siRNA was packaged in nanocarriers and administered repeatedly to a transgenic HD mouse model (*YAC 128 mice bearing the human HTT gene*). The magnitude and duration of *HTT* lowering in specific brain regions was determined as a function of intranasal dosing frequency [30]. Instead of measuring the time-course of drug concentrations in target tissue, which is the classical approach to pharmacokinetic studies, the focus was on changes in the magnitude and duration of gene expression in specific brain tissues following single and repetitive intranasal dosing [30].

**Figure 5.**
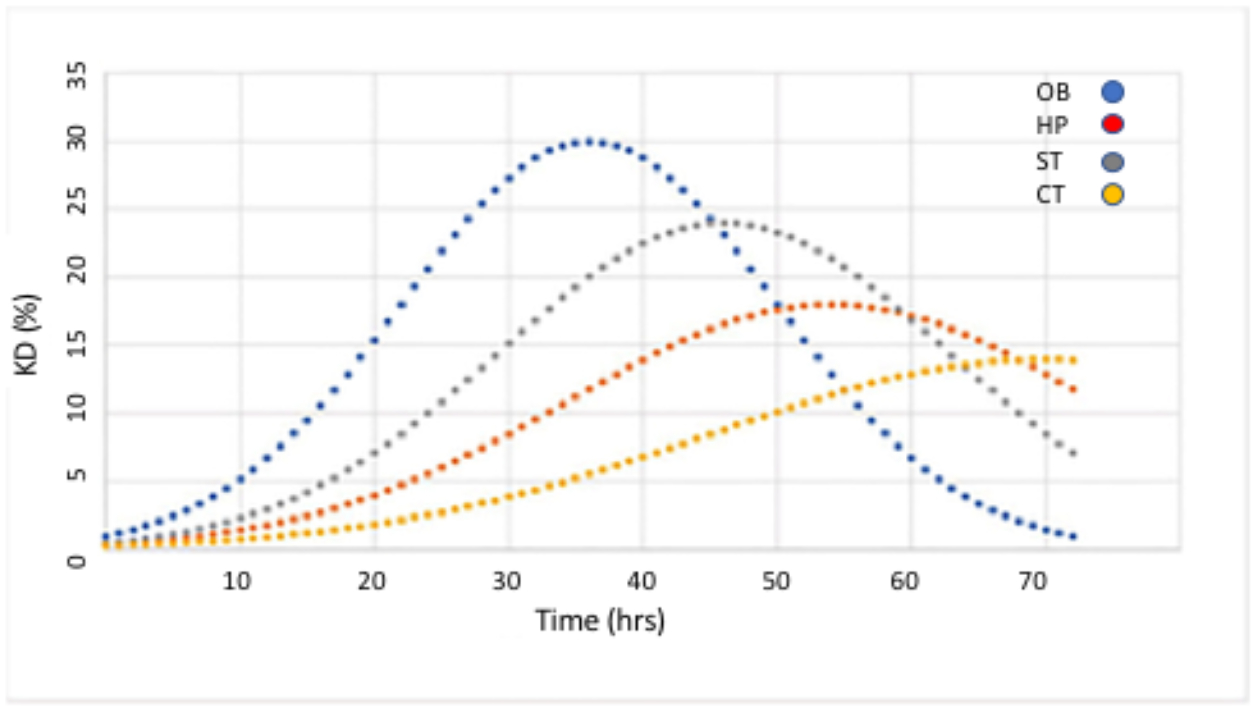
HTT lowering (KD,%) as a function of time following a single intranasal administration of a nanocarrier bearing anti-HTT siRNA in specific brain regions of YAC 128 transgenic mouse brain. OB: Olfactory Bulb; HP: Hippocampus; ST: Striatum; CT: Cerebral Cortex. Previously published by Sava et al. [30] (Copyright Elsevier, 2021).

The kinetics of the *HTT* lowering following single intranasal doses of the nanocarriers demonstrated distinct patterns, based on brain region (Figure 5). The kinetics curve of *HTT* mRNA lowering followed a “bell-shape”, with different peaks and magnitudes of effect dependent on brain region (Figure 5).

**Figure 6.**
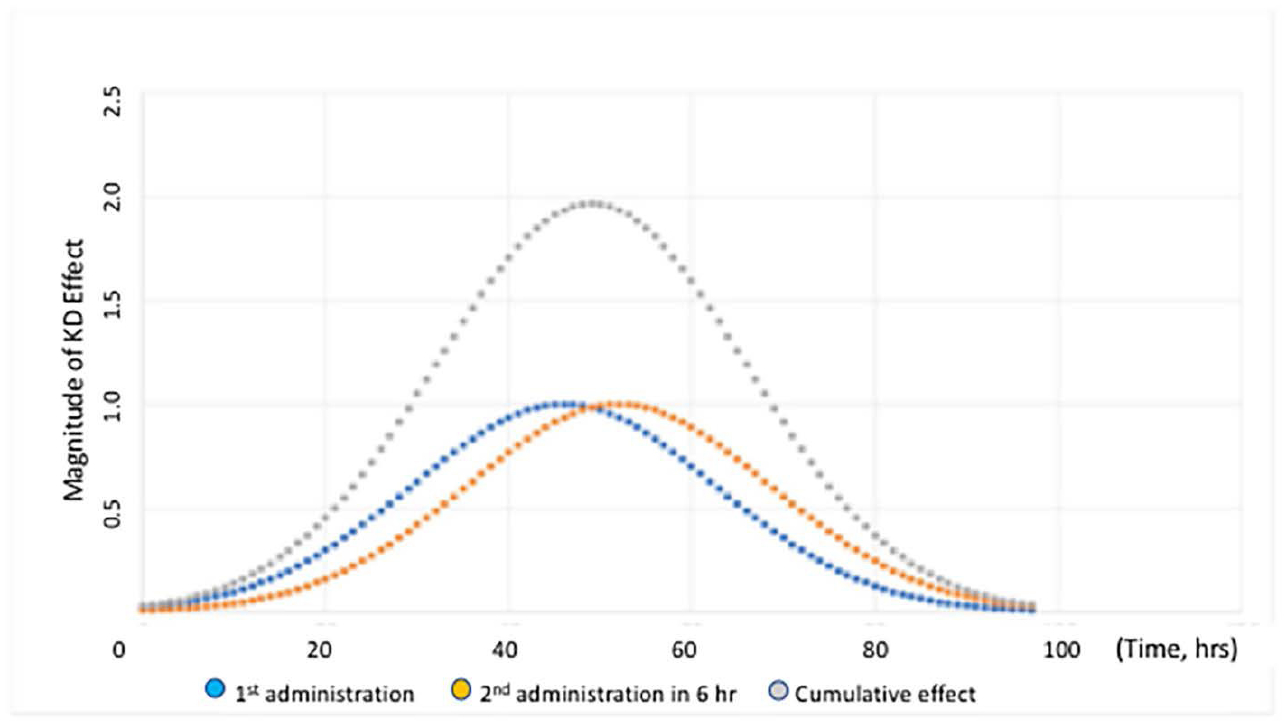
Time course of the magnitude of HTT lowering in ST of YAC 128 mice following two consecutive intranasal administrations of the nanocarriers, 6 h apart. The cumulative effect is indicated by the grey curve. The blue curve shows the kinetics following the first administration and the orange curve shows the kinetics for the second administration. Figure was previously published by Sava et al. [30] (Copyright Elsevier, 2021).

In the olfactory bulb (*OB*), the greatest extent of *HTT* lowering (*30% reduction*) was reached at 36 h. *HTT* lowering in other regions of brain required more time than in the OB. In corpus striatum (*ST*), the maximum effect was achieved at 46 hr, 10 h later than seen in OB. *HTT* lowering in hippocampus (*HP*) and cortex (*CX*) required the most time, with delays of 18 and 34 h, respectively, beyond the 36 hrs seen in OB. The magnitude *HTT* lowering was also greatest in OB with ST attaining 80% of the OB magnitude. The magnitude of *HTT* lowering in HP and CX was 60% and 47% of OB respectively. The cumulative effect on gene lowering following two consecutive intranasal administrations was determined by mathematical modeling of the kinetics data. When two consecutive doses were administered 6 hr apart, the magnitude of *HTT* lowering was doubled compared to a single administration. Moreover, the duration of *HTT* lowering was extended from 40 h to 56 h (Figure 6).

**Figure 7.**
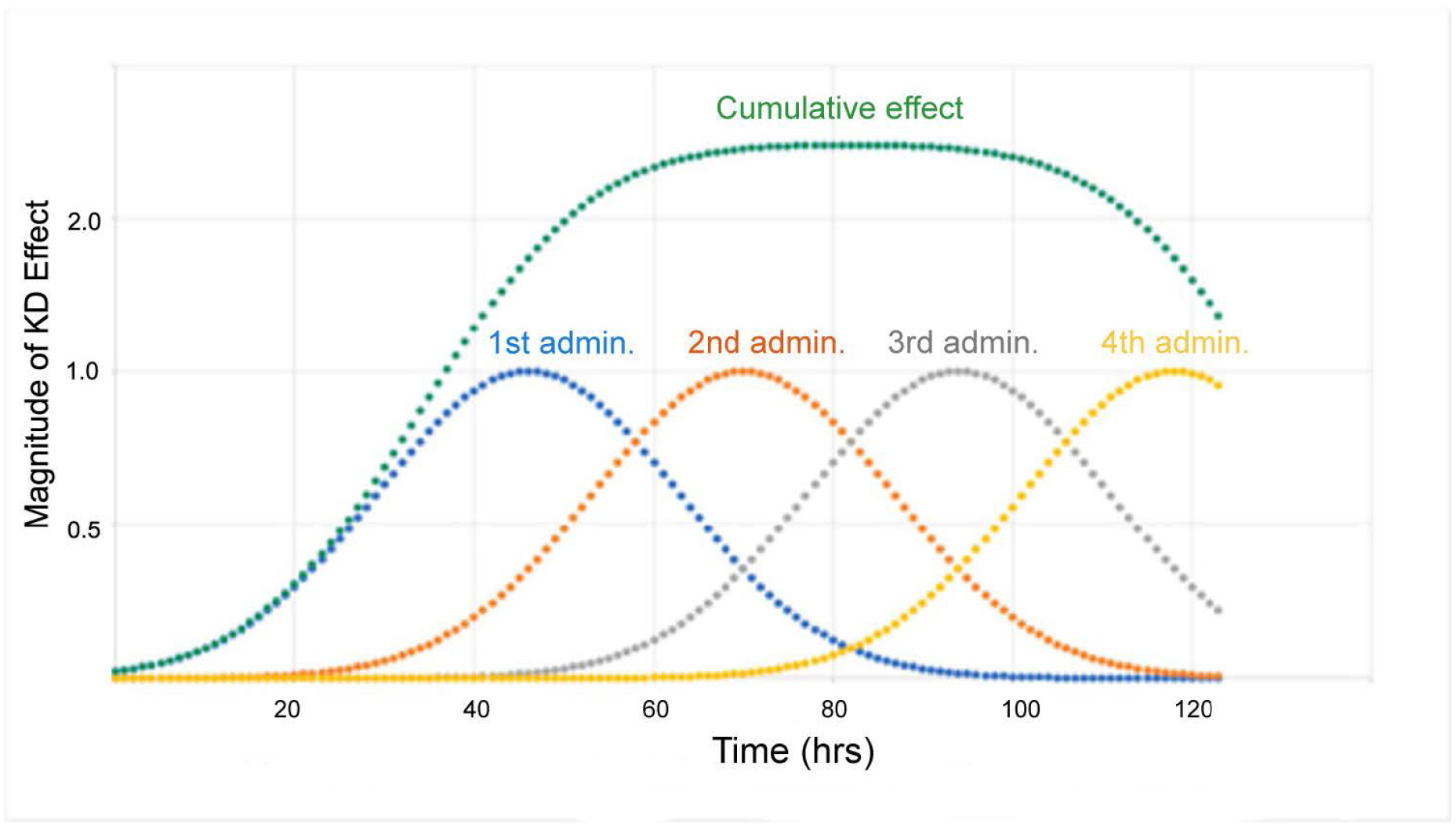
Illustration of cumulative HTT lowering in ST achieved with multiple administrations. Four consecutive administrations were made 24 h apart (blue, orange, grey and yellow curves) with green curve indicating cumulative effect. The number of administrations can be extended to prolong the duration of HTT lowering. Y-axis indicates the fold increase in KD. Figure was previously published by Sava et al. [30] (Copyright Elsevier, 2021).

The sum of the areas under the curve for each single administration equals the area under the curve of the cumulative effect. The data in Figure 6 illustrates that duration and magnitude of cumulative effect is a function of time between administrations. With minimal time between administrations, the magnitude of cumulative effect is highest. When the interval between administrations is increased beyond 45 hrs, the magnitude of effect returns to the level of a single dose administration. As an alternative, a graphical method has been utilized to model the time course of *HTT* suppression in adult female rhesus monkeys [31]. This method is not appropriate for prediction of *HTT* suppression beyond the experimental time frame because it is based on selection of a single curve from a series of similar curves generated by fitting experimental data. Determining optimal chronic dosing schedules to attain the desired steady-state level of *HTT* knockdown is more readily determined using the previously published approach (see Figure 7).

This method measures changes over time of a specific biological effect (*HTT mRNA lowering*) in various brain regions in contrast to classical pharmacokinetic studies, which measure the time course of drug concentrations over time in blood and target tissue. This approach was based on observations that parameters of gene lowering depend on multiple *dynamic* cellular physiological processes each of which occurs at distinct rates: a) transport into brain from nasal mucosa, b) distribution across brain regions, c) release of the payload (siRNA) in cells, d) clearance or metabolism of siRNA, and e) of re-synthesis of htt protein.

**Figure 8.**
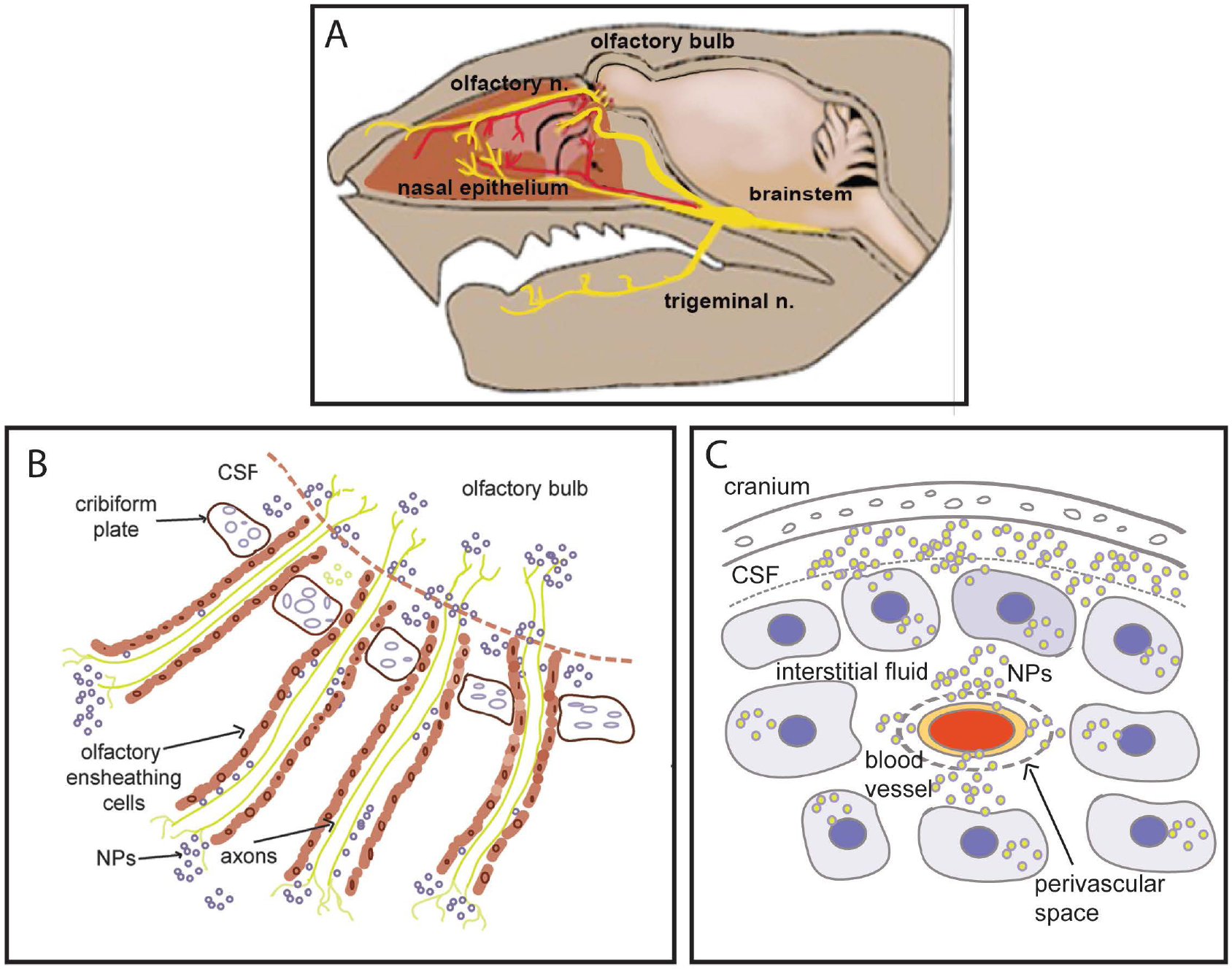
**A)** Diagram of intranasal cavity, with olfactory and trigeminal nerve endings in nasal epithelium. These nerve fibers originate in the olfactory bulb and trigeminal nucleus, respectively. **B)** Following intranasal instillation, nanocarriers can be transported directly into brain by two mechanisms: 1) Transcellular uptake into olfactory nerve terminals that transport NPs to cell bodies of the olfactory bulb and 2) Passage into the perineural space (created by the olfactory nerve ensheathing cells) which is in communication with the cerebrospinal fluid (CSF). **C)** CSF in the sub-arachnoid space percolates through the interstitial fluid which distributes the NP to cortex and hippocampus. Some NPs access CSF via the perivascular space.

The balance between extent of lowering of *HTT* expression and rate of new *HTT* expression is what dictates the frequency of dosing required to achieve a steady level of gene silencing. The distinct kinetic differences of *HTT* lowering measured in specific brain regions is due, in part, to various mechanisms of transport and distribution of nanocarriers across the brain regions from the nasal cavity (See Figure 8).

The olfactory nerves and branches from the trigeminal nerve innervate nasal epithelium. As illustrated in Figure 8, nanocarriers can be transported directly into brain, bypassing the blood-brain-barrier by two mechanisms: 1) Transcellular neural route in which nerve terminals take up the nanocarrier and transport them to cell bodies of the olfactory bulb, and 2) Passage via the perineural space (*created by the olfactory nerve ensheathing cells*) and the peri-vascular space, ultimately reaching the cerebrospinal fluid [32]. It can be inferred that transcellular transport of the nanocarriers through olfactory nerves occurs faster. The transcellular neural route is based on receptor-mediated uptake (*divalent metal transport*er) of manganese-containing nanocarriers by olfactory nerves and trigeminal nerve terminals in the nasal epithelium. Olfactory nerves extend to olfactory bulb neurons, which then project to ventral striatum and ventral pallidum via the olfactory tubercle [33]. The olfactory tubercle pathways also project to the olfactory cortex (*pyriform cortex*), located in ventral forebrain.

Slower onset of *HTT* lowering is most likely related to nanoparticle transport into the CSF via perineural and perivascular space. Nanocarriers are then distributed by bulk CSF flow and infiltration into the extracellular space where they are taken up by neurons and glial cells in cerebral cortex and hippocampus. The magnitude and duration of lowering in each brain region will also be impacted by various cellular processes: a) rates of siRNA release from nanocarriers into the target cells, b) clearance of the siRNA and c) baseline rate of *HTT* mRNA expression in the region.

The model proposed by Sava et al. [30] for determining the kinetics of gene lowering will be useful for developing an optimal dosing schedule as required for the long-term therapeutically significant gene knock-down. The ability to produce as steady and consistent level of *HTT* lowering will be essential for translation of intranasal delivery of gene therapy from animal modes to HD patients in clinical trials.

## Nanocarriers of Extended Functionality (Hybrid Nanocarriers-HNC)

Recent research has shown that successful gene therapy will require both effective gene suppression and attenuation of neuroinflammatory processes [13,34]. One approach is to utilize neuroprotective cannabinoids, such as cannabidiol (*CBD*) inserted into a lipid outer layer of the nanocarrier to create a hybrid nanocarrier (*HNC*). CBD has been reported to decrease neuronal damage and to promote growth and development of new neurons [10,35]. Cannabinoids with anti-inflammatory and anti-oxidative properties have been tested in a variety of preclinical models with promising neuroprotective benefits [36,37]. CBD has favorable pharmacological actions, but its clinical application is limited due to its poor solubility in water and reduced stability.

Liposomes have been employed in drug-delivery systems, especially for concomitant administration of hydrophilic and hydrophobic drugs. In addition to increasing the lipophilicity of the nanocarrier, a lipid shell modifies surface charge, enhances cargo protection, and improves stability [38].

The obstacles that restrict drug and gene delivery can be overcome by using liposomes and nanocarriers [39,40]. Nanocarriers that are positively charged facilitate spontaneous electrostatic interactions with nucleic acids. They also improve binding of the nanocarriers to the negatively charged components of the cell membrane to promote cellular internalization of therapeutic genes or gene-lowering molecules. Liposomes are useful because they improve gene delivery to target cells [41]. However, liposomes have limited chemical stability compared to nanocarriers made with a polymeric matrix. These nanocarriers have a longer shelf life, are more stable in biological fluids and allow safe and effective delivery of therapeutic agents to the brain [42]. In the field of cancer therapy and immunotherapy, multifunctional nanocarriers have been reported to have the additional benefit of medical visualization by many independent research groups [43–48].

**Figure 9:**
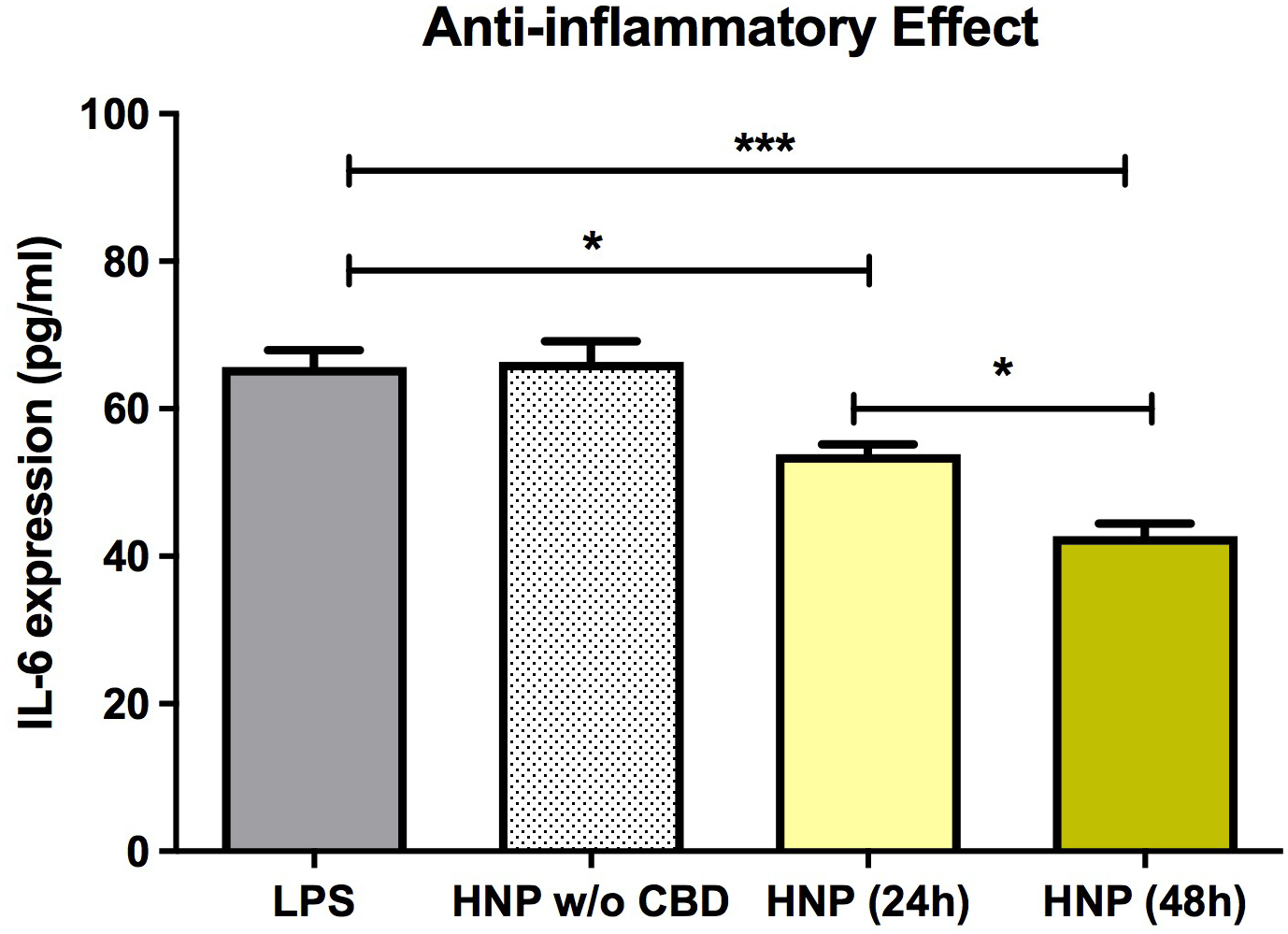
Effects of chitosan lactate-based hybrid nanoparticles or chitosan lactate-based hybrid nanoparticles loaded with cannabidiol on IL-6 expression in bone marrow mesenchymal stem cells (BMMS). Inflammation, indicated by IL-6 expression, was triggered by adding 1 μg/ml lipopolysaccharide or 1 μg/ml to the culture media. Data are expressed as mean ± standard deviation of three independent experiments. Statistical analysis was conducted using one-way analysis of variance followed by Tukey post hoc multiple comparisons test. *p < 0.05; ***p < 0.001. Abbreviations. CBD: Cannabidiol; CSL: Chitosan Lactate; HNC: Hybrid Nanocarriers; LPS: Lipopolysaccharide; SD: Standard Deviation; w/o: without. Previously published by Fihurka et al. [49] (copyright Future Medicine Ltd).

A recent report from the authors’ laboratory described the design, fabrication, and effects of hybrid nanocarriers (*HNC*) loaded with anti-*HTT* siRNA and encapsulated in a lipophilic shell containing CBD in a cell culture model of HD [49]. The HNC were shown to both lower mutant *HTT* gene expression and to attenuate inflammation in a bone marrow-derived mesenchymal stem cell line (*BMMS*). This novel HNC was optimized to allow high siRNA and CBD encapsulation and to maintain small particle size. These optimized HNC resulted in effective lowering of gene expression and minimal cell cytotoxicity. HNC with lipid shell containing CBD was effective in reducing inflammation in BMMS cell cultures (Figure 9).

The lipid lamination approach was reported by other researchers to shield chitosan-based nanocarriers and to improve the safety profile of the nanocarriers [53]. The phospholipid bimolecular membrane of the liposome simulates the mammalian cell membrane, promoting biocompatibility, and minimizing immune response and toxicity [50]. Lipophilic compounds were used in the nanocarriers because they intercalate into the phospholipid bilayer interface and displace water from the region, thereby stabilizing the lipid membrane to water hydrolysis [51–53]. CBD was used to produce the HNC to provide greater liposome stability as well as for its reported beneficial neuroprotective and anti-inflammatory activity [54–56].

**Figure 10.**
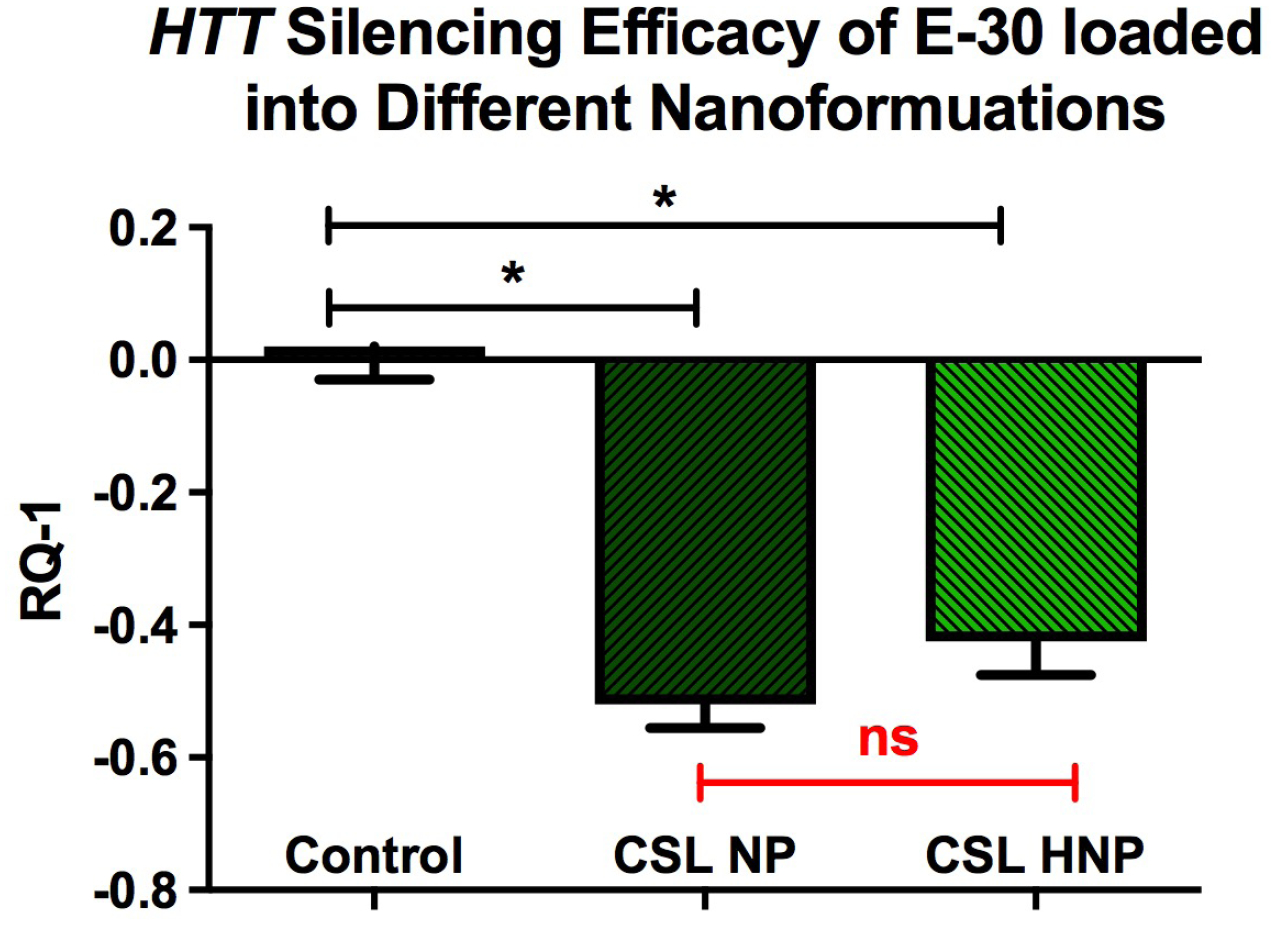
Lowering of mutant HTT following incubation of cell cultures (BMMS cells) with anti- HTT siRNAs packaged into chitosan lactate-based nanoparticles and hybrid nanoparticles. BMMS cells were plated for 48 h before treatment with siRNAs containing NPs for 24 h. Y-axis represents changes in human HTT gene expression RQ-1. Data are expressed as mean ± standard error of the mean (n=4). Statistical analysis was conducted using one-way analysis of variance followed by Tukey post hoc multiple comparisons test. ns = p > 0.05. *p < 0.05. BMMS: Bone marrow mesenchymal stem; CSL: Chitosan Lactate; HNP: Hybrid Nanoparticle; NS: Not Significant; NP: Nanoparticle. Previously published by Fihurka et al. [49] (copyright Future Medicine Ltd).

Animal studies utilizing polymeric nanocarriers have been proposed to be more effective compared to lipid-based delivery systems. HNC that exhibit characteristics of both polymeric nanocarriers and liposomes, especially with regard to physiological stability and biocompatibility, appear to be more efficacious and exhibit fewer limitations for *in vivo* drug delivery [57]. The recent report by the authors using HNC to lower *HTT* expression in BMMS cultures demonstrated slightly less lowering of mutant HTT mRNAs following the siRNA delivery with HNC compared to non- lipophilic nanocarriers (Figure 10).

Despite the obvious advantages of intranasal drug delivery, the nasal cavity presents a number of limitations for drug absorption, including low intrinsic permeability for some drugs, such as hydrophilic molecules [58]. However, the lipophilicity of HNC facilitates permeation and delivery of siRNAs through the nasal mucosa. Nasal irritation and inflammation may occur with chronic intranasal instillations, but this adverse effect can be diminished by the presence of CBD in the lipid shell of the HNP. CBD minimizes tissue injury by modulating the cytokine biology of various cell systems and decreasing inflammation [59].

## *In vivo* Trackable Intranasal Delivery of Nanocarriers to the Brain

An efficient nanocarrier system for direct nose-to-brain delivery of therapeutic molecules was developed and reported by the authors of the present review [26,60]. The nanocarrier design utilized manganese-embedded nanocarriers (*mNPs*) that target the intranasal olfactory and trigeminal nerves. This concept evolved from reports that manganese (*Mn*) particles as MnO or MnCl_2_ were actively transported into brain by the divalent metal transporter in olfactory nerves and/or through other channels (*perineural spaces around olfactory and trigeminal nerves*) [61]. Mechanisms for bypassing the BBB by the nose-to-brain delivery system have been proposed [33]. These transport routes include: 1) Direct uptake via the divalent metal transporter located on olfactory and trigeminal nerve terminals, which deliver the mNPs by axonal passage to olfactory bulb and pons, respectively; 2) Transport along the perineural spaces between axons and their peri-neural cellular sleeves, followed by distribution into the sub-arachnoid space; 3) Transport along perivascular channels. This last route of delivery was suggested to be the most likely mechanism for delivery of compacted DNA from nose to the entire neuraxis [15]. In the case of Mn particles, the transport and distribution of the metal initially followed the primary, secondary, and tertiary olfactory neurons resulting in high levels of the Mn in areas such as the olfactory bulb, the olfactory cortex, the hypothalamus, the thalamus, the hippocampus, and the habenular complex [61]. At later intervals, Mn was seen to migrate to all parts of the brain, and even into the spinal cord. Thus, the olfactory route provided a pathway for Mn which comes in contact with the olfactory epithelium to pass directly to the brain, thereby circumventing the BBB. The intranasal route of exposure was found to result in a much higher accumulation of the metal in the brain compared to intraperitoneal administration.

Nanoparticles that were to serve as carriers of therapeutic molecules were produced by amalgamation of nucleic acids (*siRNA or DNA*) with chitosan, and the Mn chelate, Mangafodipir (*MFDP*). The MFDP served to cross-link the chitosan polymer, and to ensure production of compact nanocarriers [24,26]. Physical properties of the nanocarriers were assessed by dynamic light scattering (*DLS*) and scanning electron microscopy (*SEM*). Visualization of the resulting nanocarriers using SEM showed a core–shell structure with a median dry diameter of 100 nm (*range of 90–114 nm*).

The mNPs containing anti-GFP siRNA were tested in a cell culture line that expresses GFP and were reported to significantly down-regulate GFP expression [26]. Importantly, the mNPs exhibited very low cytotoxicity. As an index of toxicity, the number of ethidium+ cells were quantified with fluorescence activated cell sorting (*FACS*). The percentage of total cells that were ethidium+ *(“dead” cells)* in mNP-treated cultures was less than those counted in lipofectamine-treated cultures, demonstrating that presence of Mn in nanocarriers in amounts used in their production was not toxic and did not impede gene silencing [26].

**Figure 11.**
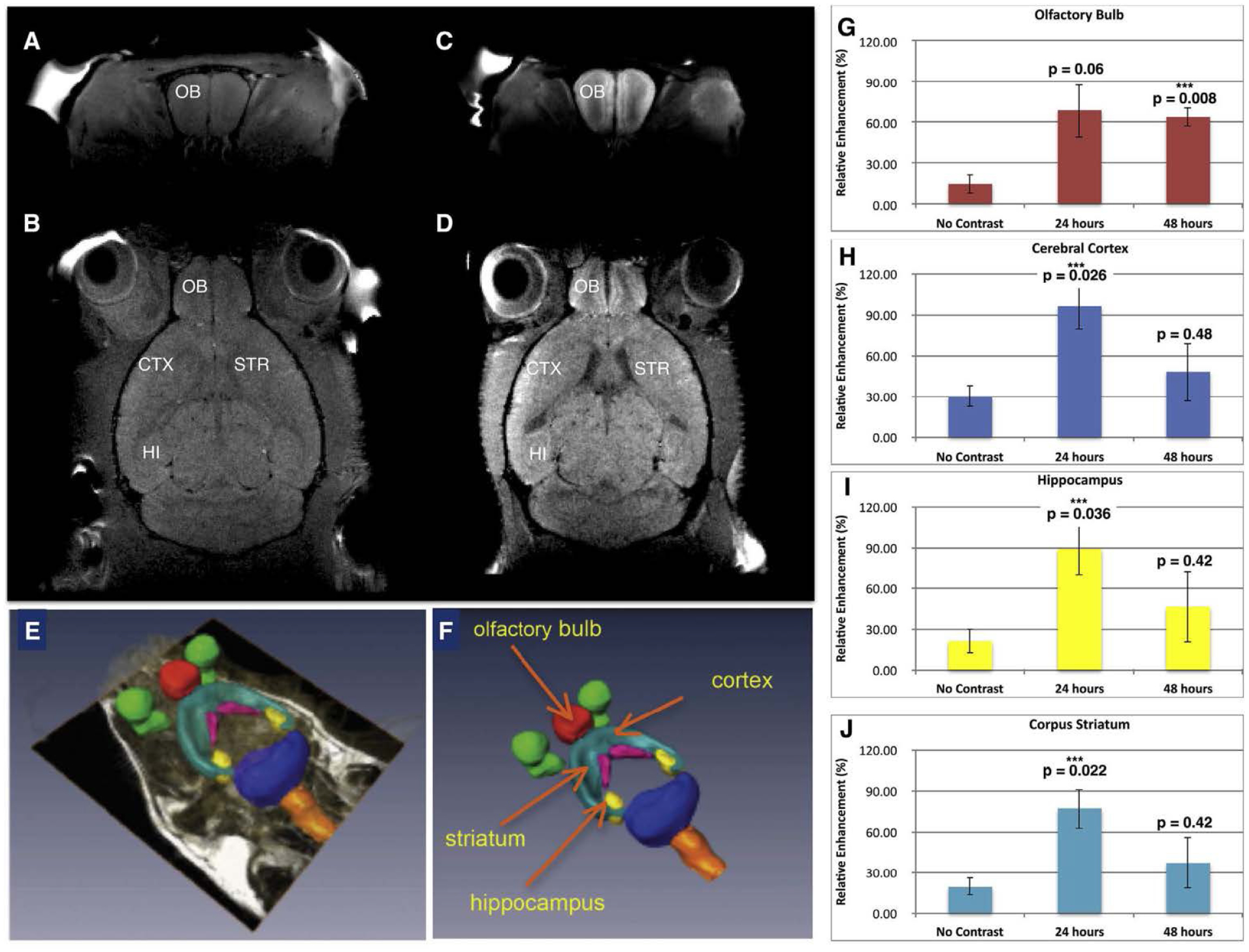
Mn-containing NPs were visualized, tracked, and quantified by MRI. **A)** Baseline T1- weighted image of coronal section through olfactory bulb and **B)** baseline horizontal section showing the anatomical regions of interest. **C)** T1-weighted MR of mouse 24 h after administration of mNPs showing enhanced Mn signal in coronal section of olfactory bulb, and **D)** T1-weighted image signal in horizontal section including olfactory bulb, cerebral cortex, striatum, and hippocampus. **E, F)** Parcellation of brain regions (to demarcate brain structures) was performed to quantify Mn signal at 24 and 48 h. **G)** Olfactory bulb; **H)** Cerebral Cortex; **I)** Hippocampus; **J)** Corpus Striatum. Mean Mn signal (±SEM, n = 3) was increased in all brain regions at 24 and 48 hrs compared to control mice (ie “no contrast”) after intranasal instillation. One-way ANOVA was performed for each brain region using Matlab Statistics Toolbox (Mathworks, Inc.). Figure was previously published by Sanchez- Ramos et al [26]; Copyright Elsevier (2018).

When mNPs carrying anti-GFP siRNA were administered intranasally to mice, the mNPs were found to accumulate in olfactory bulb and other brain regions [26]. (See Figure 11). MR imaging of anesthetized mice 24 and 48 hr after intranasal instillation of the mNPs revealed their presence in various brain regions, indicated by increased manganese signal intensity in T1- weighted images. Quantification of the Mn signal in specific brain regions utilized parcellation software. The analyzed regions (*olfactory bulb, hippocampus, cerebral cortex, and corpus striatum*) revealed significant increases in Mn signal, which peaked at 24 hr (Figure 11). The Mn signal attained the highest level in cerebral cortex, compared to other analyzed regions. However, measurements were not performed at shorter intervals after intranasal administration, so it is possible that peak Mn signal might have been higher in olfactory bulb which has direct connections to the olfactory epithelium.

The data from that study demonstrates clearly that inclusion of Mn in the nanocarrier structure is valuable for tracking the distribution of the NPs *in vivo* [26]. The report also demonstrated that Mn within the nanocarrier did not diminish functional activity of siRNA in lowering gene expression [26]. For example, eGFP mRNA expression was decreased by at least 50% in the brain regions that also exhibited significantly increased Mn signal in T1-weighted MR images.

**Figure 12.**
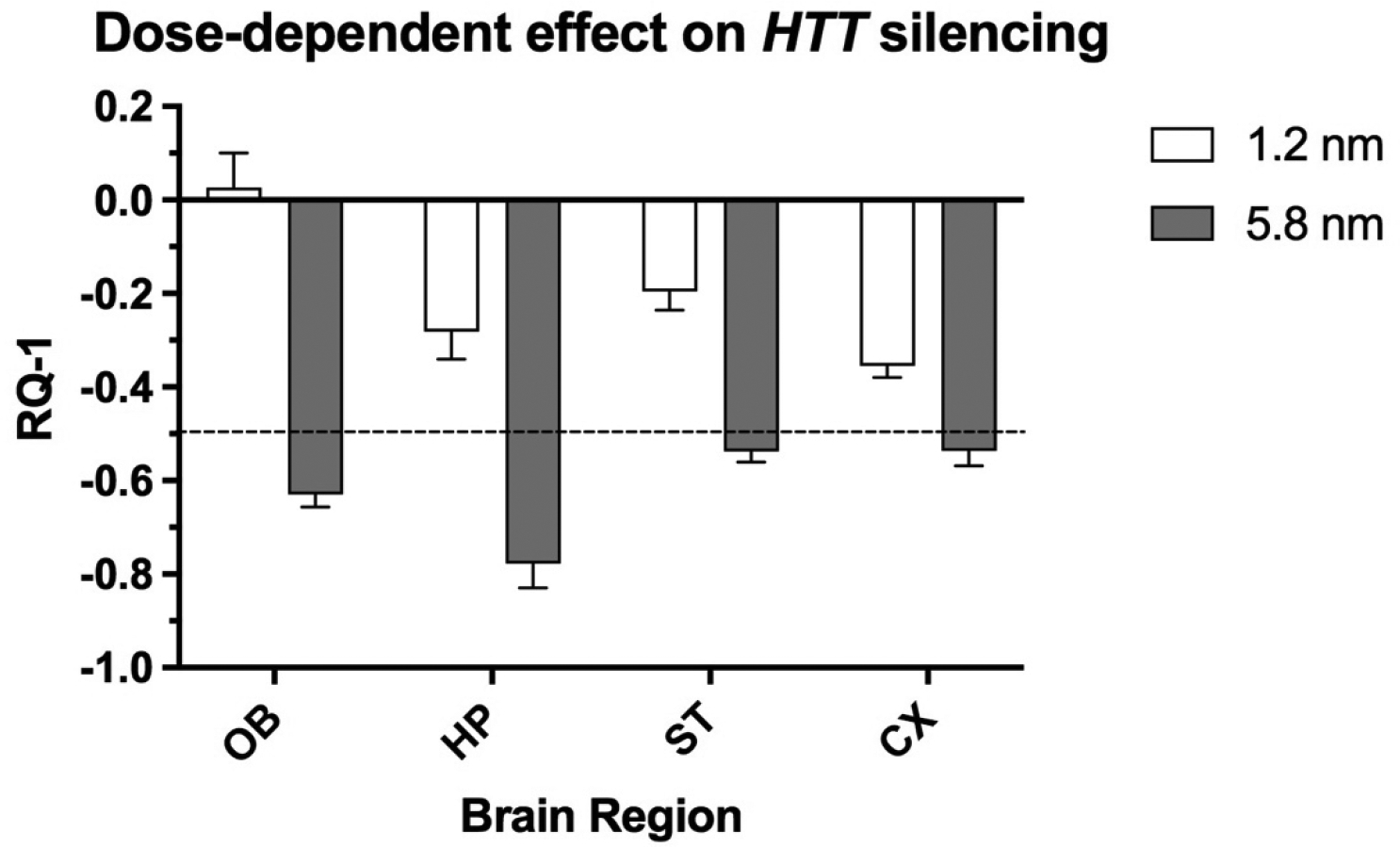
Comparison of two doses (1.2 and 5.8 nmol) of an anti-HTT siRNA (s6491) in lowering HTT mRNA expression in olfactory bulb (OB), hippocampus (HP), striatum (ST) and cortex (CX) 48h after intranasal administration to YAC128 mice. The nanocarriers loaded with anti-HTT siRNA were administered twice per day for two days, followed by euthanasia. The dashed line indicates 50% reduction of HTT expression. X-axis shows brain regions and Y-axis indicates the change in gene expression. Figure was previously published [25]; Copyright Elsevier (2020).

In addition, the intranasal instillation of NP formulations loaded with various anti-*HTT* siRNAs results in decreased expression of *HTT* mRNA in a transgenic mouse model of HD [25]. The extent of gene-lowering across brain regions was dependent on the formulation and size of the NP as well as dose of siRNA (Figure 12).

Several key factors were identified that optimize gene silencing when the siRNA is delivered by the intranasal route [25]. These factors include a) the concentration of siRNA achieved by enrichment, b) structure and lipophilicity of siRNA and c) use of a protective chitosan matrix. All but one of the siRNA structures packaged into NPs utilized a chitosan matrix. It is noteworthy that administration of “naked” siRNA by the intranasal route did not reduce brain *HTT* mRNA expression significantly, whereas direct intracerebral injection of the same “naked” siRNA was highly effective in lowering *HTT* expression [62]. Chitosan polymerizes to form a compact matrix for the nanocarrier due to electrostatic interactions between positive charged moieties of the chitosan amino groups and negative charged phosphate moieties of the siRNA structure [26]. Mangafodipir was incorporated as a crosslinking agent to stabilize the globular structure of NP and protect siRNA from degradation [60]. Another property of the chitosan NP is that it does not bind the siRNA too tightly, as recently reported, allowing release of siRNA to participate in gene silencing [21]. A further advantage of the Mn-containing NPs is the ability to track transport and distribution to brain by MRI T1-weighted imaging as was reported in earlier studies (*See* Figure 9) [26]. Chitosan is superior as a matrix for nanocarriers compared to other previously studied matrices [21]. The chitosan-based nanocarriers have the following key advantages: a) exhibit minimal or no toxicity, b) can be produced at the appropriate nano-dimensions and c) readily release nucleic acid payloads. Chitosan is biodegradable and can be digested by lysozymes produced by animals [63]. Therefore, it is practically non-toxic (*in mammals, with LD50 of 16 g/kg in rats*). Chitosan/siRNA complexes form nanocarriers [64] with a proper size around 200 nm adequate for *in vivo* delivery. Release of structurally intact siRNA from the nanocarriers, an essential prerequisite for nanocarrier-mediated RNA gene silencing was demonstrated previously [21].

A critical determinant of nanocarrier dosage forms and delivery route is the physical and chemical stability of the NP formulations [21]. Sedimentation, agglomeration or crystal growth are common physical phenomena that can impact nanocarrier stability. Decreasing particle size and increasing medium viscosity are strategies commonly applied to alleviate sedimentation problems when developing self-stabilized nanoparticle suspensions [65]. For medical applications, stable NP will be needed to deliver therapeutic doses. Common approaches to enhance chemical stability are to transform the nano-suspensions into dry solid dosage form [66] or to increase the concentration of the nanosuspensions [67].

## Summary

Nose-to-brain delivery of nanocarriers packaged with “gene-silencing” molecules is a promising alternative to more invasive routes of administration currently being applied experimentally to patients with HD. Ongoing experimental gene therapy of HD relies on invasive approaches (*intrathecal or intracerebral*) to administer ASOs to lower expression of the mutant gene. The intranasal route for gene therapy, as reviewed here, builds upon the advances in gene therapy for HD, an autosomal dominant neurodegenerative disorder. The gene-lowering effect of these agents are transient and will require chronic administration for the life-time of the HD patients. A non-invasive, safer, and equally effective approach, intranasal instillation of nanocarriers carrying gene-silencing molecules, has been developed and tested *in vitro* and *in vivo*. The design and development of nanocarriers packaged with gene-lowering agents represents a significant advance towards non-invasive nose-to-brain delivery of gene therapy for HD and other hereditary brain disorders.
